# Frequency, inequalities and spatial distribution of oral health services utilization in Peruvian children under twelve years of age: a population-based comparative analysis of the years 2017 and 2021

**DOI:** 10.1186/s12903-023-03528-4

**Published:** 2023-10-23

**Authors:** Diego Azañedo, Akram Hernández-Vásquez, Fabriccio J. Visconti-Lopez, Efrain Y. Turpo Cayo

**Affiliations:** 1 Universidad Científica del Sur, Lima, Peru; 2 Centro de Excelencia en Investigaciones Económicas y Sociales en Salud, Vicerrectorado de Investigación, Universidad San Ignacio de Loyola, Lima, Peru; 3 Sociedad Científica de Estudiantes de Medicina – UPC, Facultad de Ciencias de la Salud, Lima, Peru; 4 Universidad Nacional Agraria La Molina, Lima, Peru

**Keywords:** Child, Preschool, Health services accessibility, Dental health services, Peru

## Abstract

**Background:**

Oral diseases pose a significant public health challenge among Peruvian children. However, oral health services utilization among them is marked by inequalities, which may have been exacerbated by the COVID-19 pandemic. The objective was to compare the frequency, inequalities, determinants of inequality, and spatial distribution in oral health services utilization in Peruvian children under 12 years of age in 2017 and 2021.

**Methods:**

Comparative secondary data analysis from the Demographic and Family Health Survey (ENDES) for the years 2017 (38,787 minors) and 2021 (36,729 minors). Age-adjusted prevalence ratios (PR) with 95% confidence intervals (CI) were calculated to assess the change in oral health services utilization in the last 12 months between the years 2017 and 2021, stratifying by demographic and socioeconomic covariates. Inequality was assessed by decomposing the Erreygers Concentration Index (ECI) and calculating the percentage contribution to inequality of the independent variables. Spatial analysis was performed using spatial autocorrelation methods, Hot Spot Analysis, Cluster and Outlier analysis, Inverse Distance Weighting, ordinary Kriging, and Kulldorff analysis.

**Results:**

The probability of Peruvian children under 12 years of age using oral health services in the last 12 months was reduced by 45% in 2021 compared to 2017. The ECI identified a significant reduction in oral health services utilization inequalities at the national level (Diff: -0.0963; *p* < 0.001). The main contributor to inequality was higher tutor education level (55.2% in 2017 and 82.7% in 2021). In the comparison of spatial distribution, there was a greater dispersion of the conglomerates in which the use of oral health services is concentrated in 2021.

**Conclusions:**

The frequency of oral health services utilization in the Peruvian children under 12 years of age was halved between 2017 and 2021. This problem is transversal to the entire population at the demographic and socioeconomic level. The key factor contributing to inequalities in the utilization of oral health services was the higher educational attainment of caregivers or guardians. Despite the improvement observed in inequalities and spatial distribution of the concentration of oral health services utilization, it is necessary to keep monitoring these patterns to guide decision-making.

## Background

Oral diseases are one of the main non-communicable diseases worldwide, the most frequent being dental caries, periodontal disease, edentulism and oral cancer [1]. According to the Global Oral Health Status Report, almost half of the population (approximately 3.5 billion people) suffer from oral diseases throughout their lives. Likewise, 3 out of 4 people affected by these pathologies reside in low- and middle-income countries (LMIC) [2]. Additionally, since 1990 total disability-adjusted life years due to oral conditions have increased 64% to 16.9 million in 2015 [3]. Furthermore, LMIC have the lowest coverage and the highest socioeconomic inequality in terms of oral health care, which limits the opportunities for timely preventive and recuperative care [4, 5].

Children, especially those living in LMIC, have the highest prevalence of oral diseases in the world [1, 6, 7]. In Peru, in 2013, in the population of children affiliated with the Health Social Security (contributory insurance applicable to formal workers and their dependents), the prevalence of dental caries was 79.8% in children aged 3 to 5 years, and 90.4% in those of 12 years of age [8]. Likewise, according to the Global Oral Health Status Report, in 2019, the prevalence of untreated caries was estimated between 41.4 and 45.8% in deciduous teeth in children aged 1 to 9 years, and between 35.6 and 40.6% in permanent teeth of individuals 5 or more years of age in Peru [2]. In this sense, oral diseases in the population of Peruvian children are an important public health problem that needs urgent action on the part of the government, mainly to guarantee adequate access to free preventive and recuperative oral health treatments.

Since 2009, universal health insurance has been implemented in Peru, and given that this includes oral health care, increases in the use of oral health services and decreases in related inequality were evidenced in the period 2004–2017 [9]. Also, according to the Demographic and Family Health Survey (ENDES, by its acronym in Spanish), there is evidence of an increase over the years in the frequency of oral health services utilization in children under 12 years of age, taking into account the last six months (2014: 27.6% vs. 2019: 31.0%). However, during the COVID-19 pandemic, a decrease was reported in this percentage (2020: 19.6%) [10, 11]. Likewise, it has previously been reported that Peruvian children under 12 years of age belonging to the wealthiest quintiles, had a higher probability of using oral health services in 2019, a scenario that may have been exacerbated by the COVID-19 pandemic, increasing inequalities in oral health [12]. Finally, the pandemic has hit Peru particularly hard as it is a developing country with a limited capacity to respond to this type of event, insufficient access to basic services, high rates of poverty and unemployment [13], and spending on health that represented only 5.22% of the Gross Domestic Product in 2019, being below other countries in the Latin American and the Caribbean region and the regional average (7.88%) [14].

To date, no study assessing inequalities in oral health services utilization before and after the pandemic or the factors contributing to inequalities in this area has been conducted in Peruvian infants. Therefore, the objective of this study was to evaluate the frequency, inequalities, and determinants of inequality in the use of oral health services in children under 12 years of age, based on a comparative analysis of information from the ENDES for the years 2017 and 2021. Additionally, we carried out a comparative spatial analysis to project the distribution and clusters of oral health services utilization in the same years. Our results present an updated overview of oral health services utilization among Peruvian infants, offering a valuable foundation for the development of policies and interventions aimed at enhancing their oral health. Moreover, our study underscores the importance of obtaining more comprehensive, nationally representative data to facilitate further research on this topic.

## Methods

### Study context

In 2021, Peru had a population of approximately 33 million inhabitants. The territorial surface of Peru is 1,285,215.60 km^2^. The country’s landscape is diverse and geographically divided into 24 departments and one constitutional province (Callao). These administrative divisions are further categorized into three natural regions: the coast, the highlands, and the jungle [15, 16]. The coastal region is comprised by the departments of Lima, La Libertad, Piura, Ica, Lambayeque, Callao, Tumbes, Tacna, and Moquegua. The highland departments include Cusco, Pasco, Ayacucho, Huánuco, Junín, Cajamarca, Arequipa, Ancash, Apurímac, Huancavelica, and Puno. Finally, the jungle departments are San Martín, Ucayali, Loreto, Madre de Dios, and Amazonas [17].

The coastal region of Peru stretches along the Pacific Ocean and is home to nearly 60% of the country’s total population [18]. This region is known for its arid and desert-like landscapes, as well as for housing the majority of urban areas, including cities such as Lima, the capital of Peru. The coastal region is the country’s economic hub, foresting a diverse range of industries, international trade, and dynamic urban development. The highlands, dominated by the majestic Andes mountain range, traverse the heart of the country, defining the central part of Peru [18]. This region is distinguished by its rugged terrain, encompassing lofty plateaus at high altitudes. Moreover, it is home to nearly 30% of the Peruvian population, including numerous indigenous communities. The jungle, also known as the Amazon rainforest, covers a significant portion of Peru’s territory, primarily in the eastern part of the country. This region is renowned for its great biodiversity, dense vegetation, and ecosystems [18].

In terms of socioeconomic status, during 2020, more than 75% of individuals in rural areas experienced multidimensional poverty, whereas in urban areas, only 25% of individuals were affected [19]. Regarding the natural regions in the same year, monetary poverty in the coastal region was 25.9%, which was below the national average of 30.1% [19]. Additionally, the highlands were found to be a poorer region compared to the jungle (37.4% vs. 31.0%). However, when considering multidimensional poverty, the situation is reversed (highlands 17.3% vs. jungle 38.5%) [19]. This indicates that individuals in the jungle region may have a higher level of affordability compared to their counterparts in the highlands, but they also experience a greater degree of non-monetary deprivations [19].

### Study design and data source

This was a cross-sectional study that used information from the ENDES for the years 2017 and 2021 for comparative purposes. The ENDES is a nationally representative survey carried out annually in Peru by the National Institute of Statistics and Informatics (INEI, by its acronym in Spanish), which collects information on demographic dynamics and the health status of mothers and children under 5 years of age, associated factors with communicable and non-communicable diseases, as well as access to diagnostic and treatment services for all children under 12 years of age [20, 21].

### Sampling and data collection process

The ENDES has a complex, two-stage, probabilistic, balanced, independent sampling, stratified at the departmental level by urban and rural areas. The sampling frame is obtained from the statistical and cartographic information provided by the National Population and Housing Censuses and updated cartographic material from ENDES. The primary sampling unit at the urban level is the conglomerates and private dwellings, and, at the rural level, the rural census areas and private dwellings. The research unit for this study comprises individuals residing in both in urban and rural areas, who have spent the night prior to the application of the survey in the selected dwelling. Further specifications on the survey methodology are available in the ENDES technical reports [20, 21].

**Fig. 1:**
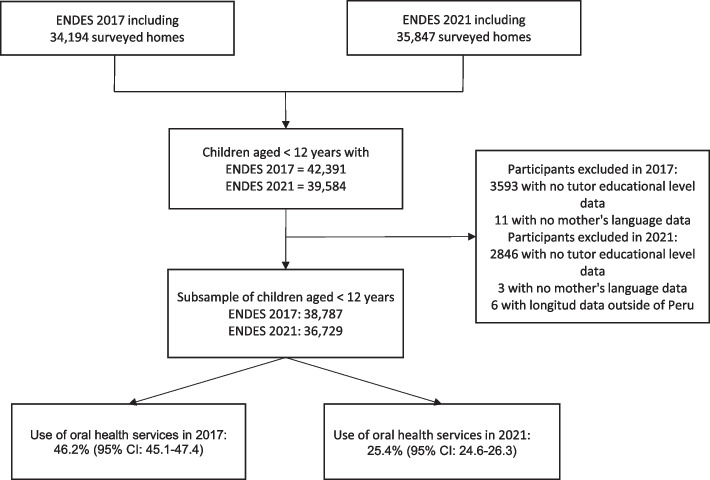
Flow chart of participants included in the study

After removing the records with incomplete information on the variables of interest for the study, the final sample consisted of 38,787 and 36,729 Peruvian children under 12 years of age in the years 2017 and 2021, respectively (See Fig. 1).

## Measures

### Outcome

The dependent variable was oral health services utilization in the last 12 months (This variable is categorical dichotomous (yes/no), constructed from the variables: QS803 (Have you ever been attended in a dental service by a dentist?); QS804U (Unit of time—How long ago was the last time you were seen in a dental service?), and QS804C (How long ago was the last service).

### Independent variable for the analysis of inequalities

Characterization of the socioeconomic level was carried out based on the home well-being index (HV271). This variable is measured continuously and is calculated in the ENDES database and follows the methodology established by the Demographic and Health Surveys Program, which is based on the characteristics of the home and household assets.

### Covariates

The following covariates were included based on previous studies, the PROGRESS framework [22], and the availability of survey variables: age (until 2 years/3 to 5 years/6 to 11 years); sex (women/men); area of residence (urban/rural); wealth index (richest/richer/middle/poorer/poorest); natural region (coast/highlands/jungle); health insurance (yes/does not have, does not know), yes is applicable when children are covered by at least one of the following types of health insurance: Integral Health Insurance (a publicly funded system primarily for the impoverished), Social Security Health Insurance (for formal employed workers and their dependents), Armed Forces and Police Health System (for employees of these institutions and their dependents), or Private insurance; mother's language (native/no native); tutor educational level (higher/secondary/until primary); and department of residence (the 24 departments and 1 constitutional province that make up the Peruvian administrative political division).

### Variables for the spatial analysis

The variables used for the spatial analysis were the latitude and longitude of the conglomerate in which the minor’s home was located. For each of these points, the number of minors with and without oral health services utilization in the last 12 months was counted. In ENDES 2017 and 2021, geolocation measurement is given by taking points in the global positioning system measured with a tablet one meter from the main door of the informant’s home. More information about the measurement of the geolocation process in the ENDES 2017 and 2021 can be found in the survey manual.

### Statistical analysis

**Fig. 2:**
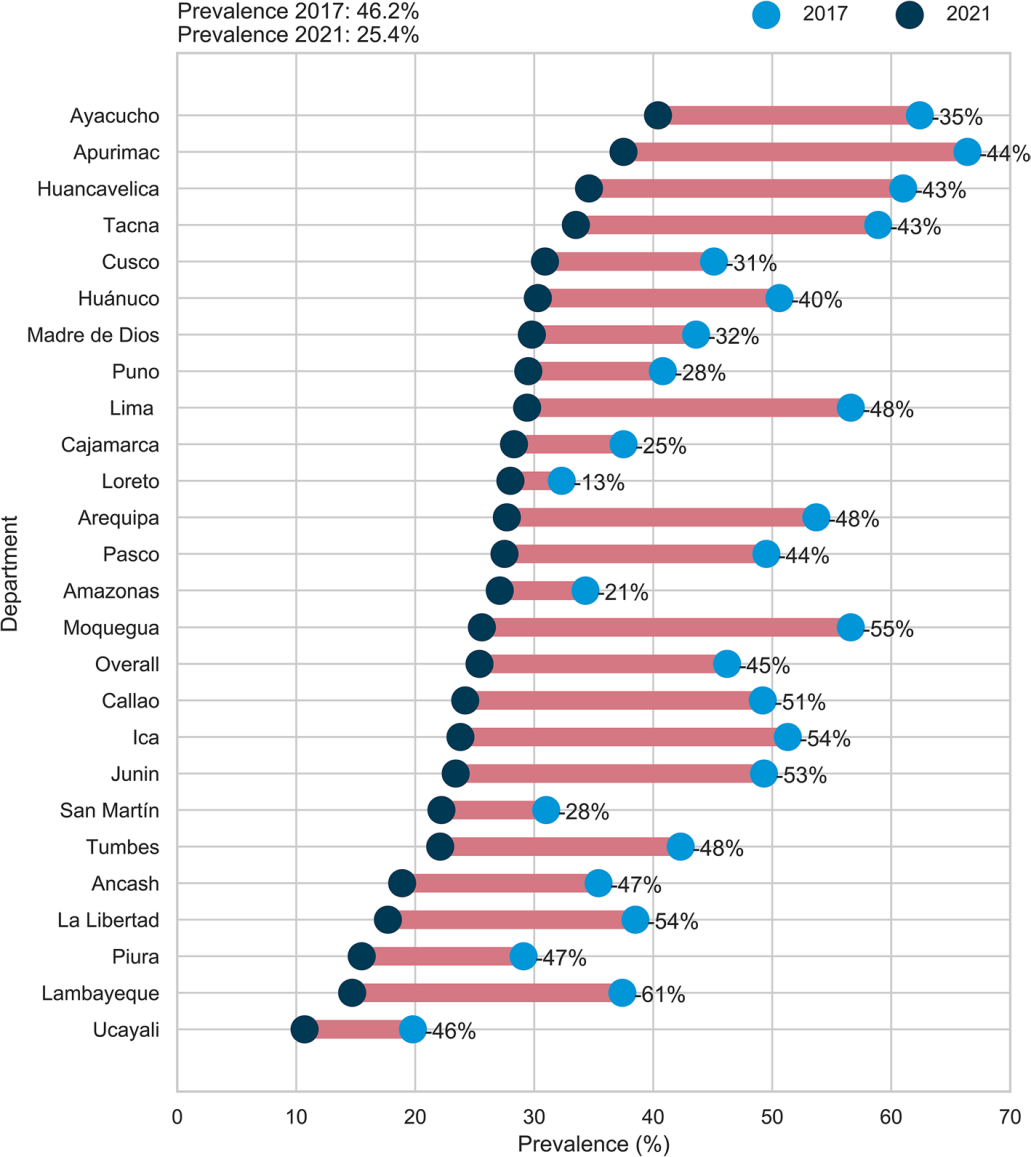
Departmental 2017–2021 differences in oral health services utilization in Peruvian children under 12 years of age

Stata 17.0 software was used for the statistical analysis (StataCorp, College Station, TX, USA) and Fig. 2 was created in Python programming language. All the analyses included the configuration of the ENDES complex sampling design, using the svy command.

A descriptive analysis of the characteristics of the sample was carried out, as well as the distribution of the outcome according to such characteristics, based on the report of absolute and relative frequencies for both years of the survey. Additionally, age-adjusted prevalence ratios (PR) were calculated (except for age when it was evaluated as an independent variable). The PRs and their 95% confidence intervals (CI) were calculated to assess the change in oral health services utilization in the last 12 months between the years 2017 and 2021, stratifying by demographic and socioeconomic covariates. In all cases, *p* values were reported, where the null hypothesis was the absence of significant differences in the frequency of the outcome between the years 2017 and 2021 for each category of the variables of interest.

The analysis of inequalities in our study employs the Erreygers Concentration Index (ECI), which was calculated using the ‘conindex’ command in Stata [23]. The ECI is a widely used measure that assesses the concentration of a certain variable of interest, such as oral health services utilization, relative to a socioeconomic variable within a population. Specifically, we utilize the ECI to evaluate the extent of inequality in oral health services utilization among Peruvian children under twelve years of age. To interpret the ECI, it is important to note that it varies in a range from -1 to 1. A value equal to 0 indicates perfect equality, indicating that oral health services utilization is distributed proportionally among all socioeconomic strata of the population. A negative value denotes a concentration of oral health services utilization among individuals with lower socioeconomic status. Conversely, a positive value indicates a concentration among individuals with higher socioeconomic status, implying inequality in the opposite direction. Additionally, a decomposition analysis of the ECI was conducted using a generalized linear model in order to establish the percentage of contribution of the independent variables to the inequality of the occurrence of the event studied. Likewise, the elasticity, ECI, and absolute and percentage contribution to inequality are reported for each independent variable. More details about the methodological aspects and interpretation of these estimates can be found in O'Donnell et al*.* [24].

Spatial analysis was conducted in accordance with the methodology outlined in a previous study [25]. The spatial autocorrelation analysis (Global Moran Index), Hot Spot Analysis (Getis-Ord Gi*), and Cluster and Outlier Analysis (Anselin Local Moran’s I) were performed using the ArcGIS Desktop version 10.5 geographic information system software (ESRI Inc., Redlands, CA, USA) [26, 27]. A total of 3163 and 3023 clusters were utilized for the years 2017 and 2021 respectively. In our study, the Moran’s Global Index assessed the overall pattern and trend of the cases that used oral health services to determine if they were clustered, scattered, or random. The Global Moran Index ranges between -1 and + 1, with positive value indicating spatial clustering, 0 indicating a randomly distributed pattern, and negative values indicating a sparse pattern. To ensure a continuous spatial distribution with values at any point in the Peruvian territory, a spatial analysis based on interpolation was carried out. The deterministic Inverse Distance Weighting (IDW) method and ordinary Kriging geostatistical method with a spherical variogram, fitted to the data with parameters obtained in R-3.6.3, were used [28, 29]. Interpolation involves predicting values at unsampled spatial locations based on the closest sampled data, resulting in estimates of oral health services utilization on a 1 × 1 km spatial resolution grid [30]. Lastly, using the SaTScan V10.1 software (Martin Kulldorff, Boston, MA, USA), a Kulldorff spatial exploration analysis (Bernoulli model) was used to identify clusters with a high concentration of oral health services utilization [31]. This analysis uses the creation of a circular window that scans the area of influence of the study, where the radius of the circle depends on the maximum size of the population considered for the clusters. A maximum of 25% was set, that is, a group can contain a maximum of 25% of the total population with cases of oral health services utilization. The results are reported as a log-likelihood ratio (LLR) and *p*-value, where it is interpreted that the risk within the window is different from that outside the window. The p-value is calculated through the simulation model of Monte Carlo with 999 replicates; a low *p*-value suggests that the identified cluster is significant and probably not the result of chance. If the LLR value is positive and high it indicates a possible spatial concentration of cases [32]. The SaTScan output for statistically significant clusters includes the location of the center of the scan window, the radius of the scan window, and the *p*-value of the cluster [32]. Statistically significant clusters were defined as those with a *p* < 0.05.

### Ethical considerations

The conduct of the study did not require the approval of an ethics committee as it was an analysis of aggregated secondary data that is in the public domain and does not allow the identification of the evaluated participants.

## Results

A total of 38,787 and 36,729 children under 12 years of age were included in the years 2017 and 2021, respectively. In both years (2017 and 2021), a higher frequency of participants was identified in the age group of 6 to 11 years (50.2%; 51.3%), male sex (50.7%; 50.9%), the urban area of residence (74.1%; 77.1%), the poorer wealth quintile (24.1%; 23.5%), the coastal region (56.3%; 57.8%), those with health insurance (83.8%; 88.7%), children with mothers with non-native languages (81.6%; 83.8%) and those with mothers who have secondary education level (45.9%; 47.1%) (See Table 1).

**Table 1:** Characteristics of the Peruvian children under 12 years included in the ENDES 2017 and 2021

Characteristic	2017 (*n* = 38787)		2021 (*n* = 36729)	
	**n**	**% (95% CI)**	**n**	**% (95% CI)**
Age				
Until 2 years	12724	24.8 (24.3—25.3)	11641	22.5 (22.0—22.9)
3 to 5 years	10923	24.9 (24.4—25.5)	11064	26.2 (25.6—26.7)
6 to 11 years	15140	50.2 (49.6—50.9)	14024	51.3 (50.7—51.9)
Sex				
Female	19273	49.3 (48.5—50.0)	18156	49.1 (48.3—49.9)
Male	19514	50.7 (49.9—51.5)	18573	50.9 (50.1—51.7)
Area of residence				
Urban	26580	74.1 (72.6—75.5)	24050	77.1 (76.4—77.8)
Rural	12207	25.9 (24.5—27.4)	12679	22.9 (22.2—23.7)
Wealth index				
Richest	3400	13.7 (12.4—15.1)	3260	14.9 (13.9—15.9)
Richer	5464	17.8 (16.8—18.9)	4959	18.2 (17.2—19.1)
Middle	7635	20.4 (19.3—21.4)	6765	20.5 (19.6—21.4)
Poorer	10626	24.1 (22.8—25.3)	9682	23.5 (22.5—24.5)
Poorest	11662	24.1 (22.7—25.5)	12063	23.0 (22.1—23.9)
Region				
Coast	15784	56.3 (54.1—58.6)	14851	57.8 (56.7—59.0)
Highlands	12246	26.9 (25.0—28.8)	11947	25.4 (24.3—26.7)
Jungle	10757	16.8 (15.3—18.4)	9931	16.7 (15.8—17.6)
Health insurance				
Yes	33286	83.8 (83.0—84.6)	33414	88.7 (88.0—89.4)
Does not have/does not know	5501	16.2 (15.4—16.9)	3315	11.3 (10.6—11.9)
Mother’s language				
Native	9011	18.4 (17.2—19.7)	8459	16.2 (15.3—17.0)
No native	29776	81.6 (80.3—82.8)	28270	83.8 (82.9—84.7)
Tutor educational level				
Until primary	10806	25.7 (24.3—27.0)	8800	21.2 (20.3—22.1)
Secondary	17573	45.9 (44.6—47.3)	17359	47.1 (45.9—48.2)
Higher	10408	28.4 (27.1—29.8)	10570	31.8 (30.7—32.8)

*CI* Confidence interval

^a^All estimates took into account the ENDES sample design

The probability of oral health services utilization in the last 12 months in the population of Peruvian children under 12 years of age was reduced by 45% in 2021 compared to 2017. A reduction in the probability of oral health services utilization was observed between the years compared for all the categories of the characteristics evaluated (See Table 2). The greatest reduction in the probability of oral health services utilization (55%) was identified in children of the middle wealth quintile, those living in the coastal region (50%), and children with mothers who have a secondary (50%) or primary education level (50%) (See Table 2).

**Table 2:** Frequencies and prevalence ratios of access to oral health services during the last year in Peruvian children under 12 years of age between 2017 and 2021

Characteristic	2017 (*n* = 38787)		2021 (*n* = 36729)		PR (95% CI)	*p*-value
	**No**	**Yes**	**No**	**Yes**		
	**n (%)**	**n (%)**	**n (%)**	**n (%)**		
Overall	22449 (53.8%)	16338 (46.2%)	27913 (74.6%)	8816 (25.4%)	0.55 (0.53—0.57)	< 0.001
Age						
Until 2 years	9601 (73.6%)	3123 (26.4%)	10,018 (86.5%)	1623 (13.5%)	0.51 (0.47—0.56)	< 0.001
3 to 5 years	6087 (54.4%)	4836 (45.6%)	8064 (73.6%)	3000 (26.4%)	0.58 (0.55—0.61)	< 0.001
6 to 11 years	6761 (43.7%)	8379 (56.3%)	9831 (69.9%)	4193 (30.1%)	0.53 (0.51—0.56)	< 0.001
Sex						
Female	11092 (52.9%)	8181 (47.0%)	13766 (73.8%)	4390 (26.2%)	0.55 (0.52—0.58)	< 0.001
Male	11357 (54.6%)	8157 (45.5%)	14147 (75.4%)	4426 (26.6%)	0.54 (0.51—0.56)	< 0.001
Area of residence						
Urban	14673 (50.0%)	11907 (49.9%)	18239 (73.6%)	5811 (26.4%)	0.52 (0.50—0.55)	< 0.001
Rural	7776 (64.5%)	4431 (35.5%)	9674 (77.8%)	3005 (22.2%)	0.62 (0.57—0.68)	< 0.001
Wealth index						
Richest	1549 (39.9%)	1851 (60.1%)	2163 (63.1%)	1097 (36.9%)	0.60 (0.55—0.66)	< 0.001
Richer	2770 (45.9%)	2694 (54.0%)	3617 (70.6%)	1342 (29.5%)	0.54 (0.50—0.59)	< 0.001
Middle	4108 (50.4%)	3527 (49.6%)	5214 (77.4%)	1551 (22.6%)	0.45 (0.41—0.49)	< 0.001
Poorer	6309 (57.4%)	4320 (42.6%)	7494 (77.8%)	2188 (22.2%)	0.51 (0.48—0.55)	< 0.001
Poorest	7716 (66.7%)	3946 (33.3%)	9425 (79.4%)	2638 (20.6%)	0.62 (0.56—0.68)	< 0.001
Region						
Coast	8778 (50.6%)	7006 (49.4%)	11626 (75.1%)	3225 (24.9%)	0.50 (0.47—0.53)	< 0.001
Highlands	6371 (52.9%)	5875 (47.0%)	8614 (73.1%)	3333 (26.9%)	0.57 (0.53—0.61)	< 0.001
Jungle	7300 (65.7%)	3457 (34.3%)	7673 (75.2%)	2258 (24.9%)	0.72 (0.66—0.78)	< 0.001
Health insurance						
Yes	18746 (52.2%)	14540 (47.8%)	25192 (74.1%)	8222 (25.9%)	0.54 (0.51—0.56)	< 0.001
Does not have/does not know	3703 (61.9%)	1798 (38.1%)	2721 (78.7%)	594 (21.3%)	0.53 (0.47—0.60)	< 0.001
Mother’s language						
Native	5084 (55.5%)	3927 (44.5%)	6337 (76.2%)	2122 (23.8%)	0.53 (0.49—0.58)	< 0.001
No native	17365 (53.4%)	12411 (46.6%)	21576 (74.3%)	6694 (25.7%)	0.55 (0.52—0.57)	< 0.001
Tutor educational level						
Until primary	7139 (65.3%)	3667 (34.7%)	7220 (82.7%)	1580 (17.3%)	0.50 (0.45—0.55)	< 0.001
Secondary	10300 (54.3%)	7273 (45.7%)	13394 (76.6%)	3965 (23.4%)	0.50 (0.48—0.53)	< 0.001
Higher	5010 (42.5%)	5398 (57.5%)	7299 (66.3%)	3271 (33.7%)	0.58 (0.54—0.61)	< 0.001

*PR* Prevalence ratios indicates the comparison of the year 2021 with respect to 2017 for each category of the variables of interest, *95% CI* 95% confidence interval

^a^All estimates took into account the ENDES sample design and were adjusted for age, except for age when evaluated as the independent variable

The ECI identified significant reductions of oral health services utilization inequality at the population level in Peruvian children under 12 years of age (Diff: -0.0963; *p* < 0.001). Significant reductions in the ECI were also identified in all age groups, in males and females, in the area of rural residence, the highlands and jungle region, the insured and uninsured, native and non-native mother tongue, and the secondary and primary educational levels (See Table 3).

**Table 3:** Changes in inequalities measured by means of the concentration index of access to oral health services during the last year in Peruvian children under 12 years of age between 2017 and 2021

Characteristic	2017		2021		Diff	*p*-value*
	**n**	**ECI**	**n**	**ECI**	**2021–2017**	
Overall	38787	0.2203	36729	0.1239	-0.0963	< 0.001
Age						
Until 2 years	12724	0.2084	11641	0.0626	-0.1457	< 0.001
3 to 5 years	10923	0.2359	11064	0.1107	-0.1252	< 0.001
6 to 11 years	15140	0.2344	14024	0.1606	-0.0737	< 0.001
Sex						
Female	19273	0.2345	18156	0.1323	-0.1022	< 0.001
Male	19514	0.2068	18573	0.1163	-0.0905	< 0.001
Area of residence						
Urban	26580	0.1657	24050	0.1406	-0.0250	0.157
Rural	12207	0.1924	12679	0.0957	-0.0967	< 0.001
Region						
Coast	15784	0.1808	14851	0.1646	-0.0162	0.465
Highlands	12246	0.1969	11947	0.1414	-0.0554	0.030
Jungle	10757	0.2765	9931	0.1368	-0.1397	< 0.001
Health insurance						
Yes	33286	0.2157	33414	0.1221	-0.0936	< 0.001
Does not have/does not know	5501	0.2940	3315	0.1501	-0.1439	< 0.001
Mother’s language						
Native	9011	0.1784	8459	0.0448	-0.1336	< 0.001
No native	29776	0.2355	28270	0.1403	-0.0951	< 0.001
Tutor educational level						
Until primary	10806	0.1657	8800	0.0414	-0.1243	< 0.001
Secondary	17573	0.1222	17359	0.0155	-0.1067	< 0.001
Higher	10408	0.1109	10570	0.1393	0.0283	0.313

*ECI* Erreygers concentration index, *Diff* differences between ECI 2017 and ECI 2021

^*^p-value indicates the probability of non-significant change in inequalities according to the concentration index from 2017 to 2021

^a^All estimates took into account the ENDES sample design

The main contributor to inequality in both years was the higher education level. In 2017, the higher education contribution percentage was 55.2%, with rural residency as the second largest contributor at 24.7%. In 2021, the majority of the inequality was explained by higher education, comprising 82.7%, while the age group of 3 to 5 years age group contributed 4.8% (See Table 4).

**Table 4:** Decomposition analysis of inequalities of access to oral health services during the last year in Peruvian children under 12 years of age in 2017 and 2021

Characteristic	2017				2021			
	***n* = 38787**				***n* = 36729**			
	**Elasticity**	**ECI**	**Absolute contribution**	**Percentage contribution (%)**	**Elasticity**	**ECI**	**Absolute contribution**	**Percentage contribution (%)**
Age								
Until 2 years	Ref				Ref			
3 to 5 years	0.0520	0.0147	0.0031	1.4	0.0408	0.0366	0.006	4.8
6 to 11 years	0.1632	-0.0179	-0.0117	-5.3	0.1064	-0.0196	-0.0083	-6.7
Sex								
Female	Ref				Ref			
Male	-0.0089	0.0051	-0.0002	-0.1	-0.0078	0.0041	-0.0001	-0.1
Area of residence								
Urban	Ref				Ref			
Rural	-0.0209	-0.6512	0.0545	24.7	-0.0016	-0.6763	0.0044	3.6
Region								
Coast	Ref				Ref			
Highlands	0.0100	-0.3053	-0.0122	-5.6	0.0128	-0.313	-0.016	-12.9
Jungle	-0.0126	-0.4402	0.0223	10.1	0.0064	-0.4119	-0.0105	-8.5
Health insurance								
Yes	Ref				Ref			
Does not have/does not know	-0.0201	0.1143	-0.0092	-4.2	-0.0071	0.0949	-0.0027	-2.2
Mother’s language								
Native	Ref				Ref			
No native	-0.029	0.0897	-0.0104	-4.7	0.0008	0.0752	0.0002	0.2
Tutor educational level								
Until primary	Ref				Ref			
Secondary	0.0524	-0.0004	-0.0001	0.0	0.043	-0.0487	-0.0084	-6.8
Higher	0.0656	0.4637	0.1217	55.2	0.0625	0.4099	0.1025	82.7
Residual			0.0625				0.0568	

*ECI* Erreygers concentration index

^a^All estimates took into account the ENDES sample design

At the departmental level, it was detected that the greatest percentage reduction in the use of oral health services between 2021 and 2017 occurred in Lambayeque, with 61%, followed by Moquegua, with 55%, and both, Ica and La Libertad with 54%. On the other hand, the departments that experienced the lowest percentage reductions were Loreto, with 13%, Amazonas, with 21%, and San Martín and Puno, with 28% (See Fig. 2).

**Fig. 3:**
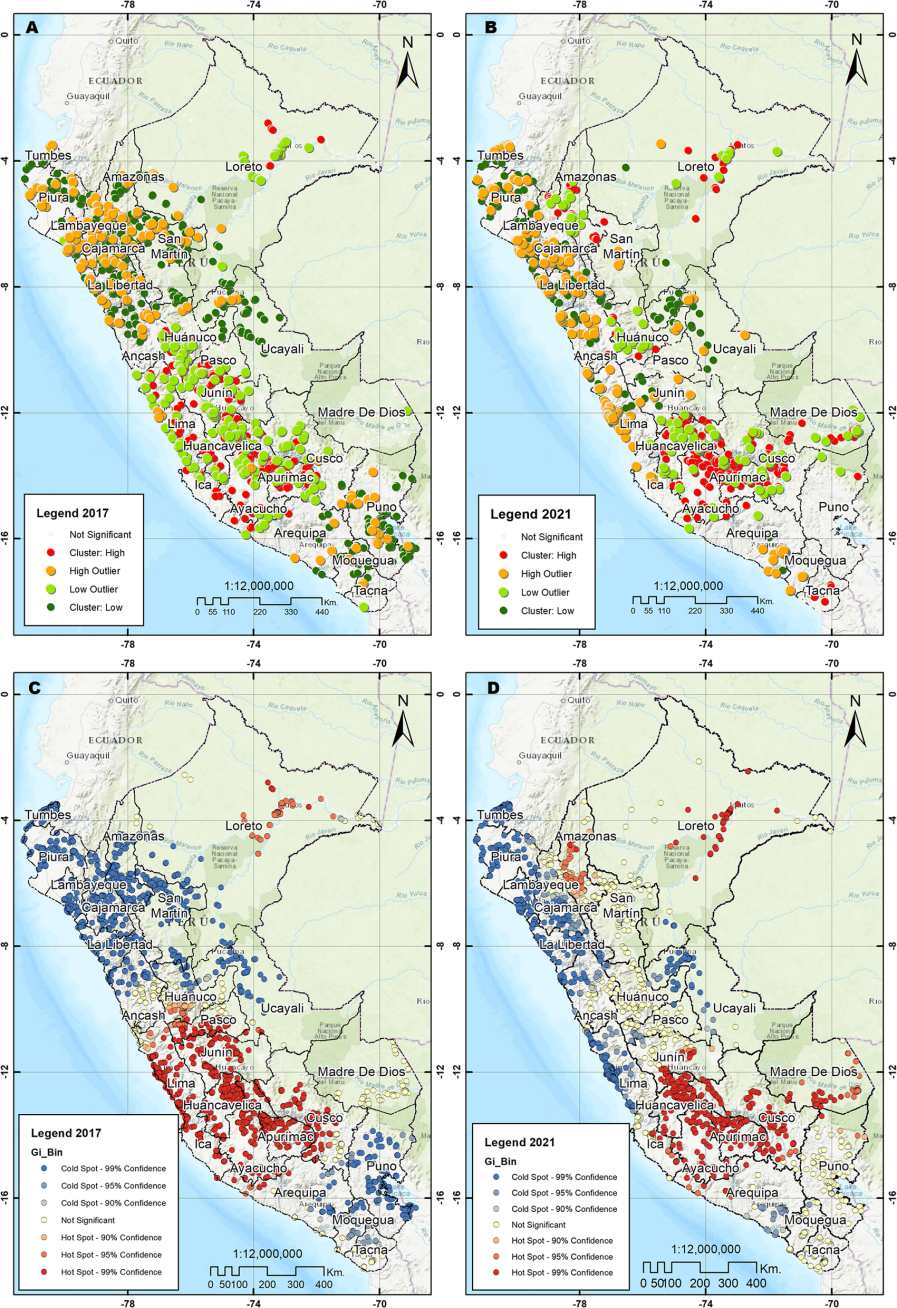
Spatial distribution of oral health services utilization using Local Moran and Hot Spot analysis. **A** Cluster and outlier analysis, 2017. **B** Cluster and outlier analysis, 2021. **C** Hot spot analysis Getis Ord Gi, 2017. **D** Hot spot analysis Getis Ord Gi, 2021

**Fig. 4:**
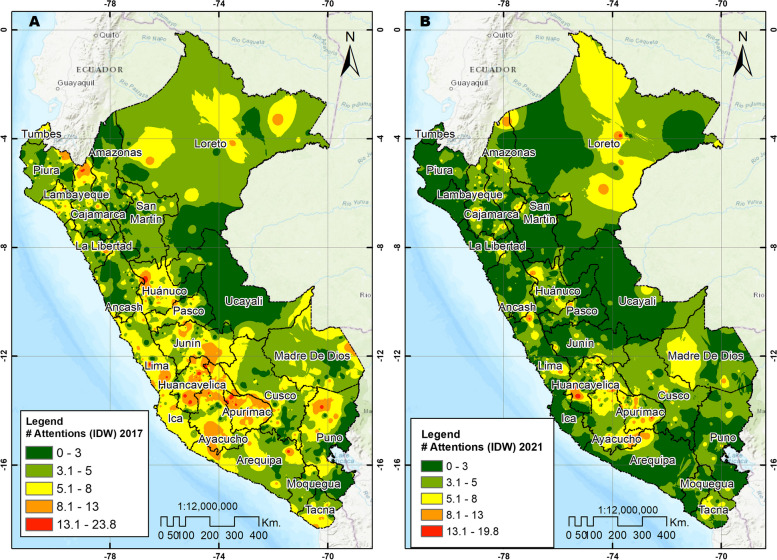
Spatial distribution of oral health services utilization using spatial interpolation IDW. **A** Interpolation IDW, 2017. **B** Interpolation IDW, 2021

**Fig. 5:**
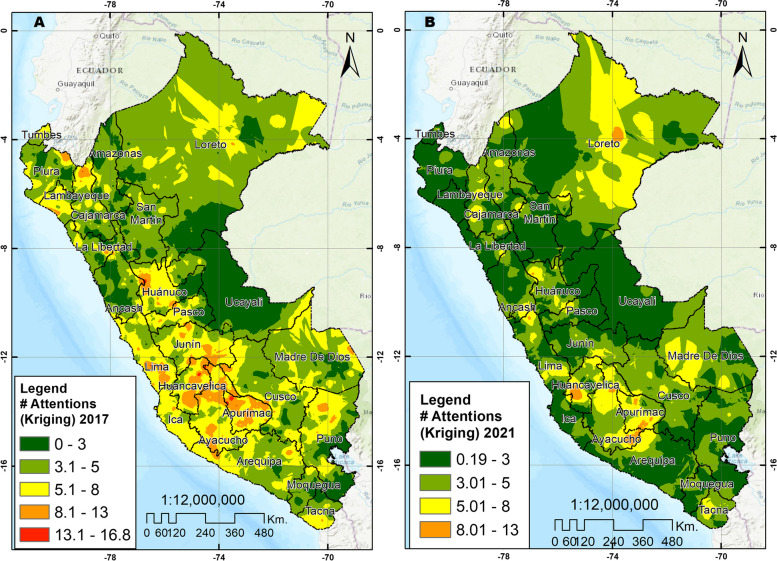
Spatial distribution of oral health services utilization using spatial interpolation ordinary spherical Kriging. **A** Interpolation ordinary spherical Kriging, 2017. **B** Interpolation ordinary spherical Kriging, 2021

**Fig. 6:**
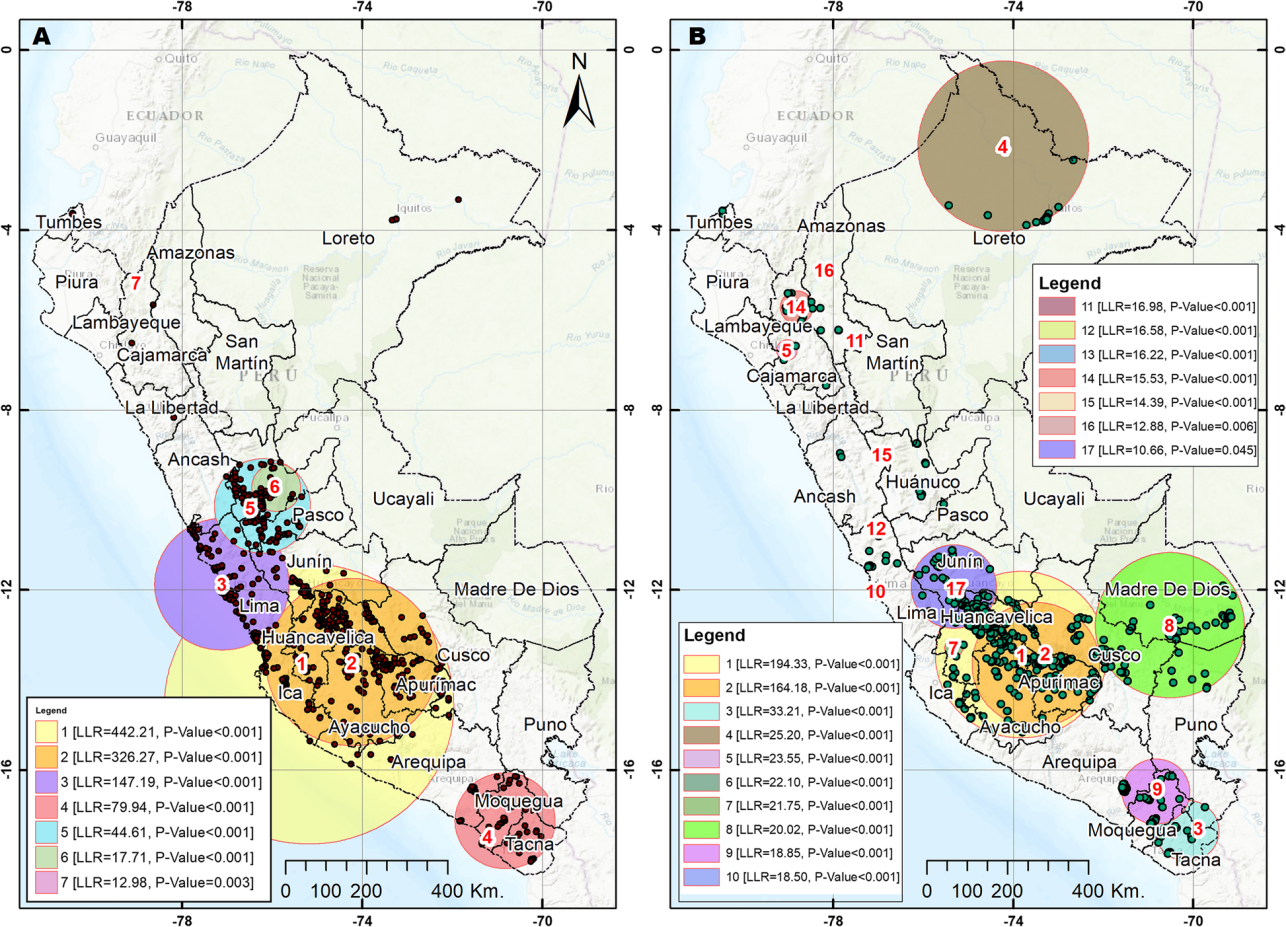
Spatial distribution of oral health services utilization using spatial SaTScan analysis. **A** Spatial SatScan analysis, 2017. **B** Spatial SatScan analysis, 2021

Spatial autocorrelation of the clusters in the two years was positive and statistically significant in 2017 (Moran’s Index 0.0649, *p* < 0.001 and z-score 9.93) and 2021 (Moran’s Index 0.0878, *p* < 0.001 and z-score 16.21). In 2017, clusters with a high concentration of use of oral health services were identified in Loreto, as well as in departments of the central and southern highlands, such as Huánuco, Pasco, Junín, Huancavelica, Ayacucho, Apurímac, Cusco and Arequipa and the departments of Lima and Ica. In 2021, the departments of Huánuco, Huancavelica, Apurimac, Ayacucho and Cusco maintained conglomerates with a high concentration of oral health services. Likewise, unlike in 2017, clusters with a high concentration were identified in the departments of Puno, Tacna, Madre de Dios, Amazonas, San Martín, and Ancash (See Fig. 3). The analyses of the spatial distribution of oral health services utilization (IDW interpolation, spherical Kriging interpolation and SaTScan analysis) showed consistent patterns of cluster grouping with a high concentration of use of oral health services, in comparison with the Moran local analysis and Hot Spot analysis (See Figs. 4, 5, and 6). When spatially comparing oral health services utilization in the study years, a greater dispersion of the conglomerates in which the use of oral health services was concentrated was found in 2021. According to SaTScan analysis, the statistically significant clusters increased from 7 to 17 between 2017 and 2021, respectively. According to the Hot spot analysis, the department of Amazonas and Madre de Dios had a particular change in the increase in oral health services utilization in 2021 compared to 2017.

## Discussion

In 2021, the probability of oral health services utilization by Peruvian children under 12 years of age decreased by 45% compared to 2017. A similar trend of reduced oral health services utilization has been identified in some ecological studies, encompassing the majority of municipalities in Brazil as well [33, 34]. This problem could be due to the fact that during the COVID-19 pandemic the World Health Organization issued a series of recommendations to reduce the application of aerosol-generating procedures in the context of oral health care as a measure to prevent the spread of the virus [35]. Consequently, at the national level, the Peruvian Ministry of Health restricted face-to-face care in cases of oral urgencies or emergencies in the public and private spheres, limiting the opportunities for primary prevention in the dental office [36]. Another contributing factor was the economic and labor uncertainty, which affected many families throughout Latin America during the pandemic [37]. This may have forced the prioritization of expenses (e.g., food, housing, and transportation) and the use of health insurance, leaving oral health aside, even after vaccination and the lifting of care restrictions. As a result of unemployment, the reduction in family income and the increase in food prices also brought with it a scenario of food insecurity, which affected Peru to a greater extent compared to other Latin American countries [38, 39]. Likewise, due to the fear of contagion, especially due to the high rate of excess mortality in Peru [40], it is possible that both patients and dentists postponed treatments or preventive visits [41–44].

Our results show that at the national level, there was a reduction in inequality in oral health services utilization between 2017 and 2021. This could be due to the increase in public health coverage since the implementation of universal health insurance in 2009, which is publicly funded with no cost to the poor [45]. Likewise, greater coverage of conditional transfer programs over the years should be highlighted, such as JUNTOS, which reaches districts with a high percentage of poverty [46], as well as the efforts to integrate the promotion of oral health in programs such as the control of growth and development (CRED, by its acronym in Spanish) of girls and boys under five years of age, implemented in 2017, and of mandatory application in public and private health establishments nationwide [47]. All these measures may have generated, from different fronts, a positive impact on the inequalities in oral health services utilization among different population groups. Nevertheless, the relatively diminished inequality in oral health services utilization between the study years appears contradictory, considering the economic repercussions of the COVID-19 pandemic, particularly on the socioeconomically disadvantaged segment of the population. This paradoxical phenomenon might be attributed to changes in patterns of oral health services utilization, resulting from pandemic-related restrictions. For instance, a study utilizing data from the Public Health System of Brazil reported a nationwide decline in the total number of oral health procedures between March and August of 2020, with more pronounced reductions observed in both individual and collective preventive procedures [33]. Unfortunately, the ENDES lacks information regarding the purpose of dental visits, impeding a specific evaluation. Nonetheless, we hypothesize that individuals with higher socioeconomic status may have sought oral health services less frequently for preventive or cosmetic purposes, while those with lower socioeconomic standing, who may have poorer overall health, may have sought dental care due to genuine emergencies or urgent needs more frequently. Indeed, another study conducted in Brazil, reported greater reductions in urgent dental care during the most stringent period of the pandemic in municipalities with higher human development indices compared to 2019 [34]. In the Peruvian context, the pandemic scenario could have created a temporary and deceptive reduction in inequalities in oral health services utilization. Further research is warranted to investigate and substantiate our hypothesis.

The higher educational level of the legal guardian was the main contributing factor to the inequality in oral health services utilization in children under 12 years of age. This could be explained in that, with more education, guardians have greater knowledge and understanding of the importance of oral health care for the general health of their children [48, 49]. Additionally, guardians with a higher level of education often have higher incomes, health insurance, and better financial security, allowing them to pay for quality dental services for their children [50]. These differences in knowledge, economic capacity, and access to information can result in a gap in oral health services utilization among children whose parents have a higher level of education. Therefore, it is important to address these inequalities through strategies, such as parent education programs through schools or the health system, which could help improve their knowledge and understanding of the importance of oral health care in their minors and how to access oral health services in the national territory [51].

On the other hand, the largest percentage reductions in oral health services utilization occurred in the coastal departments (Lambayeque, Moquegua, Ica and La Libertad) and the smallest reduction were observed in the jungle (Loreto, Amazonas and San Martín). This may be because the coastal regions had higher mortality figures during the COVID-19 pandemic compared to the jungle regions [52], and this may have generated fear of exposure to SARS-CoV-2 in residents of the coast, and consequently, a reduction in health care, including oral health care. In this regard, although a decrease in oral health services utilization has been observed across all departments, it is imperative to prioritize actions and allocate resources to implement and strengthen strategies that promote the utilization of oral health services, particularly in the departments most significantly affected. On the other hand, in the spatial analysis, it was evidenced that, compared to 2017, a greater number of regions were found with conglomerates with a high concentration of oral health services utilization in the year 2021, denoting a greater dispersion of the conglomerates in which oral health services utilization in the last year under evaluation was concentrated. This could be considered favorable since the concentration of use would no longer only occur in some areas of the national territory. On the other hand, this may be related to the percentage reductions in oral health services utilization identified in our study, which in 2017 may have been concentrated in some regions, mainly Lima, Ica and the central highlands, and, given that, during the pandemic, care was reduced to urgencies or emergencies throughout the territory, a dispersion on the use of services can be seen, probably because routine and/or preventive face-to-face care was not carried out during confinement. However, these results should be considered exploratory, and further spatial investigations and monitoring of concentration patterns are warranted in the future to inform decision-making processes.

The main limitation of our study is its cross-sectional design, which prevents the establishment of cause-and-effect relationships. In addition, there is the possible introduction of recall bias since the outcome of interest was assessed through self-reported data in the past. In addition, there is the possibility of social desirability bias, as respondents could have modified their answers to fit the standards of their peers. Also, this study performed an analysis of secondary data, limiting the scope of the evaluation to the variables available in the survey. As a result, it hindered the assessment of more insightful aspects of oral health services utilization patterns, such as the intensity of utilization or the specific reasons for dental attendance. As for the spatial analysis, the results were obtained according to the ENDES sampling conglomerates in which the respondents’ dwelling was located, since the ENDES reports the geographic coordinates in this way to preserve the identity of the participants, which could limit the accuracy of the spatial distribution. Despite these limitations, this study provides information on the frequency, inequalities, and spatial distribution on oral health services utilization in Peruvian children under 12 years of age, using the most up-to-date information available, and calculating estimates with national representativeness. Understanding of Peru’s socio-economic and geographic context is crucial for comprehending the challenges and opportunities related to access to oral health services. By acknowledging the country’s diverse regions, including the coast, highlands, and jungle, researchers and policymakers can develop context-specific approaches to improve oral health outcomes and ensure equitable access to oral healthcare throughout Peru.

## Conclusions

Our findings show a worrying decrease in the frequency of oral health services utilization in the population of Peruvian children under 12 years of age between 2017 and 2021. The reduction was generalized in all the categories of characteristics evaluated, indicating a problem that affects all population groups. It is important that the Peruvian government reflects on these results and take measures to improve oral health services utilization in the child population, especially in the most vulnerable groups, such as economically disadvantaged children, who continue to be affected by inequalities in this area to date. Therefore, it is essential to monitor and strengthen the existing upstream strategies designed to increase oral health service utilization in Peruvian children. Additionally, we identified a reduction in inequalities regarding oral health services utilization and an improvement in the spatial distribution of this outcome at the national level. However, these patterns are likely influenced by pandemic-related restrictions, and further research is necessary to gain a better understanding of these paradoxical trends in dental attendance after the pandemic, which can inform decision-making processes. It is highly recommended that future studies thoroughly investigate the impact of the pandemic and inequalities considering the reasons for oral health services utilization as the main outcome. In light of this, we urge the responsible entities in Peru tasked with providing nationally representative data to prioritize the collection of more comprehensive information for oral health research.

## Data Availability

The data supporting the findings of this study are publicly available from the National Institute for Statistics and Informatics (INEI) website (https://proyectos.inei.gob.pe/microdatos/). The databases can be obtained by entering the “Query by surveys” tab, selecting in “Survey” the option “Demographic and Family Health Survey – ENDES”, for the years 2017 and 2021.

## Abbreviations

LMIC: Low and middle-income countries
ENDES: Demographic and Family Health Survey
INEI: National Institute of Statistics and Informatics
PR: Prevalence ratios
CI: Confidence interval
ECI: Erreygers concentration index
IDW: Inverse distance weighting
LLR: Log-likelihood ratio
